# Synthesis of Poly (2‐hydroxyethyl ethyleneimine) and Its Mucoadhesive Film Formulations When Blended with Chitosan for Buccal Delivery of Haloperidol

**DOI:** 10.1002/mabi.202400642

**Published:** 2025-03-03

**Authors:** Sitthiphong Soradech, Adrian C. Williams, Vitaliy V. Khutoryanskiy

**Affiliations:** ^1^ Reading School of Pharmacy University of Reading Whiteknights Reading RG6 6AD UK; ^2^ Expert Centre of Innovative Herbal Products Thailand Institute of Scientific and Technological Research Pathum Thani 12120 Thailand

**Keywords:** buccal drug delivery, chitosan, miscibility, mucoadhesive films, poly(2‐hydroxyethyl ethyleneimine)

## Abstract

Mucoadhesive films are attractive for buccal drug delivery because of their extended retention on the mucosal surface, enabling sustained drug delivery to and across the tissue. In this study, poly(2‐hydroxyethyl ethyleneimine) (P2HEEI) was synthesized by reacting linear polyethyleneimine (L‐PEI) with 2‐bromoethanol and combined with chitosan to formulate mucoadhesive films for buccal delivery of haloperidol. The polymer displayed excellent solubility in water, a low glass transition temperature (−31.6 °C) and low toxicity in human dermal skin fibroblast cells. This polymer was then blended with chitosan before films were formed by a casting technique. Differential scanning calorimetry and scanning electron microscopy confirmed that chitosan and P2HEEI were fully miscible in the blends. The films based on chitosan‐P2HEEI blends were more elastic and had enhanced mechanical properties. Films containing haloperidol were also formulated. The release of haloperidol from the films increased as the P2HEEI content in the blends was raised. Mucoadhesion of these films on ex vivo sheep buccal mucosal tissues was evaluated using a tensile method. All films were mucoadhesive but increasing P2HEEI content in the blend gradually reduced adhesion to the buccal mucosa.

## Graphical abstract

Mucoadhesive chitosan‐poly(2‐hydroxyethyl ethyleneimine) (P2HEEI) films were developed for buccal delivery of haloperidol. P2HEEI is synthesized by reacting linear polyethyleneimine with 2‐bromoethanol, achieving high solubility, low glass transition temperature, and biocompatibility. The films exhibit enhanced mechanical properties, tunable mucoadhesion, and controlled drug release. Increasing P2HEEI content improved elasticity and drug release but reduced  mucoadhesion. These findings highlight the potential of blend composition to optimize buccal drug delivery.

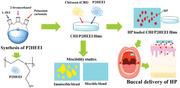

## 1 Introduction

Transmucosal drug delivery is commonly used to administer drugs to and through the buccal mucosa to provide local or systemic effects^[^ ^1^ ^]^ and has generated interest as an alternative to oral drug delivery. The advantages of the buccal route include avoidance of gastrointestinal acid‐related hydrolysis, rapid onset of action, increased patient compliance (particularly those with  dysphagia), and a wide variety of drugs and excipients exist that cause little or no mucosal damage or irritation.^[^ ^2^, ^3^, ^4^ ^]^ Mucoadhesive films are commercially available and can extend drug contact time with the mucosa, enabling direct delivery of the drug to or through the tissue.^[^ ^2^, ^5^ ^]^

Chitosan is a natural polysaccharide synthesized by the de‐acetylation of chitin.^[^ ^6^ ^]^ It has many applications, such as in nutrition products, drug delivery systems, cosmetics, artificial skin, food packaging, because of its biocompatibility, biodegradability, mucoadhesive and antimicrobial properties, and its film forming ability.^[^ ^7^, ^8^, ^9^, ^10^ ^]^ Furthermore, chitosan films have been used in wound care.^[^ ^11^, ^12^ ^]^ Chitosan is also beneficial for buccal drug delivery due to its ability to enhance drug penetration by opening tight junctions and increasing the paracellular permeability of mucosal membranes. This polymer can also control drug release from buccal drug delivery systems and has excellent mucoadhesive properties.^[^ ^13^ ^]^ The mucoadhesive properties of chitosan are predominantly due to the interaction between its net positive charges and the negatively charged mucosal surface.^[^ ^12^, ^13^ ^]^ However, employing chitosan alone in mucoadhesive films is limited due to their brittleness, reflected in chitosan's relatively high glass transition temperature (T_g_≈131 °C).^[^ ^14^, ^15^ ^]^

The characteristics of chitosan films can be enhanced by blending with other polymers.^[^ ^16^ ^]^ Polymer mixing provides a simple and cost‐efficient approach to develop novel systems with desired properties. Previously, mucoadhesive films containing chitosan were prepared in combination with cellulose ethers.^[^ ^16^ ^]^ Blends of chitosan have also been reported with poly(N‐vinyl pyrrolidone),^[^ ^17^ ^]^ poly(ethylene oxide),^[^ ^18^, ^19^ ^]^ and poly(vinyl alcohol)^[^ ^20^ ^]^ to improve physicochemical properties. Luo et al.^[^ ^13^ ^]^ developed mucoadhesive polymeric films from chitosan blends with hydroxyethylcellulose (HEC). While blending chitosan with HEC improved the mechanical properties of materials, mucoadhesion of films to buccal mucosa decreased as the HEC content in the blends increased. Abilova et al.^[^ ^14^ ^]^ reported the formulation of the films by blending chitosan with poly(2‐ethyl‐2‐oxazoline) and demonstrated their potential application in ocular drug delivery. Remuñán‐López et al.^[^ ^21^ ^]^ developed a bilayer oral drug delivery film using chitosan for controlled release and ethylcellulose to prevent drug loss in saliva. This system facilitated unidirectional drug delivery with optimized swelling and release properties. Subsequently, Koland et al.^[^ ^22^ ^]^ formulated buccal films containing ondansetron hydrochloride using chitosan and PVP K30, demonstrating prolonged drug release and enhanced bioavailability. Abruzzo et al.^[^ ^23^ ^]^ introduced chitosan/gelatin films for propranolol hydrochloride delivery, emphasizing their mucoadhesive properties and prolonged in vivo drug release. Further advancements included the use of supercritical solution impregnation for drug‐loaded chitosan films, as shown by Tang et al.,^[^ ^24^ ^]^ which enhanced drug loading capacity and controlled the release of ibuprofen. Tejada et al.^[^ ^25^ ^]^ developed chitosan‐based buccal films incorporating miconazole nitrate, which exhibited enhanced antifungal activity against *Candida* spp.

Linear polyethyleneimine (L‐PEI) is a polymer comprising two methylene (‐CH_2_CH_2_‐) groups and a secondary amino group in each repeating unit. It can be synthesized by hydrolysis of poly(2‐ethyl‐oxazolines) (PEOZ).^[^ ^26^, ^27^ ^]^ L‐PEI has a semi‐crystalline structure^[^ ^28^, ^29^ ^]^ and a glass transition temperature around −29.5 °.^[^ ^30^ ^]^ L‐PEI can dissolve in water only at high temperatures^[^ ^28^ ^]^ and forms a gel at room temperature.^[^ ^31^ ^]^ Additionally, L‐PEI is known to be cytotoxic^[^ ^30^, ^32^ ^]^; the limited solubility and toxicity of L‐PEI are major concerns when evaluating its suitability for pharmaceutical and biomedical applications.^[^ ^33^ ^]^ Derivatization of L‐PEI can increase its water solubility and/or decrease its toxicity. Patil et al.^[^ ^34^ ^]^ synthesized hydroxyethyl substituted linear polyethyleneimine (HELPEI) using nucleophilic substitution reaction to produce non‐toxic polymer for siRNA delivery. Previously, we modified L‐PEI by reaction with 3‐bromo‐1‐propanol to form poly(3‐hydroxypropyl ethyleneimine). This polymer, abbreviated as P3HPEI, also had a low glass transition temperature (T_g_ = −38.6 °C). Polymeric films were prepared based on the blends of chitosan with P3HPEI, and were used for rapid release of haloperidol. However, at higher doses (5 mg mL^−1^), cell viability of P3HPEI was less than 80%.^[^ ^15^ ^]^ This is due to the longer alkyl chain in P3HPEI, which increases the toxicity of modified L‐PEI in human dermal skin fibroblasts.

Here, we synthesized poly(2‐hydroxyethyl ethyleneimine) or P2HEEI to blend with chitosan as a mucoadhesive film platform for buccal delivery of haloperidol. P2HEEI with a high degree of hydroxyethyl substitution was synthesized by reaction of linear polyethyleneimine with 2‐bromoethanol. The physicochemical and toxicological properties of this polymer were assessed before blending with chitosan to prepare novel elastic and mucoadhesive films for buccal delivery of haloperidol. Miscibility between polymers, mucoadhesive properties, and haloperidol release from these films were studied.

## 2 Results and Discussion

### 2.1 Synthesis and Characterization of Poly (2‐hydroxyethyl ethyleneimine), P2HEEI

**Figure 1:**
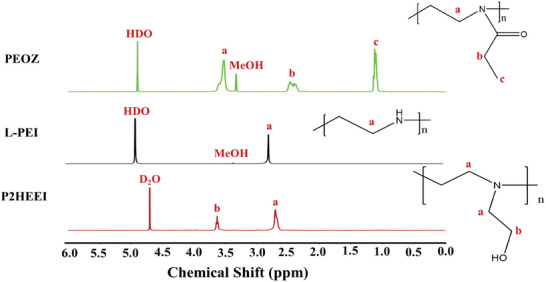
^1^H‐NMR spectra of PEOZ and L‐PEI were recorded in MeOH‐d_4_, and P2HEEI spectrum was recorded in D_2_O.

L‐PEI was produced by hydrolyzing PEOZ, effectively removing all amide groups. The conversion of PEOZ into L‐PEI was evaluated spectroscopically using ^1^H‐NMR and FTIR. As it is seen in **Figure** **1**, two PEOZ signals from the side moieties ≈2.44 and 1.13 ppm, disappeared, and the signal from the two CH_2_ groups in the backbone shifted to 2.75 ppm. FTIR (Figure S1, Supporting Information) also confirmed complete hydrolysis of the amide bonds in PEOZ through the loss of the carbonyl vibrational mode at 1626 cm^−1^ and the appearance of new strong bands at ≈1474 and 3263 cm^−1^ due to the N‐H of PEI. These ^1^H‐NMR and FTIR results correlate well with other reports.^[^ ^26^, ^35^ ^]^ L‐PEI was subsequently alkylated with 2‐bromoethanol in absolute ethanol with potassium carbonate used as a base. The structure of the resulting P2HEEI was confirmed spectroscopically with ^1^H‐NMR and FTIR (Figure 1; Figure S1, Supporting Information). Two signals in the ^1^H‐NMR spectrum of P2HEEI, at 2.62 ppm (signal a) due to the methylene groups in the backbone and the CH_2_ in the side group adjacent to nitrogen and at 3.60 ppm (signal b) due to the methylene group (CH_2_) adjacent to the hydroxyl group (‐OH) at the side moiety are consistent with the new material. A downfield shift of signal b was caused by a de‐shielding effect of the ‐OH group. The signal from the CH_2_ group on the side group adjacent to the nitrogen overlapped with the signal from the CH_2_ groups of the backbone.

**Figure 2:**
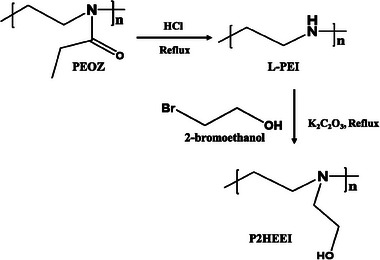
Scheme of chemical transformations from PEOZ through L‐PEI to form P2HEEI.

P2HEEI was synthesized with varying molar ratios of 2‐bromoethanol (0.02 to 0.06 moles to 1 unit‐mole of L‐PEI repeating unit) under reaction times of 24 or 48 h. **Figure** **2** shows the reaction scheme from PEOZ through L‐PEI to P2HEEI.

Figure S2 (Supporting Information) and **Table** **1** summarize ^1^H NMR spectra and the degrees of substitution (DS) of the materials. The results show that increasing 2‐bromoethanol from 0.02 to 0.06 molar ratios and reacting for 24 h increased the DS from 59.2% to 84.3%. The effect of reflux time on the DS was also evaluated using the molar ratio of L‐PEI to 2‐bromoethanol of 0.02: 0.06. A longer time to reflux (24 to 48 h) resulted in a higher DS, increasing from 84.3% to 97.1%. FTIR (Figure S1, Supporting Information) also indicated successful chemical modification of L‐PEI derivative with hydroxyethyl groups to form P2HEEI from a new broad absorption peak at 3414 cm^−1^ due to an OH‐ stretching mode.

**Table 1:** Degrees of substitution of hydroxyethyl polyethyleneimine prepared at different molar ratios of L‐PEI: 2‐bromoethanol: base and reflux time.

L‐PEI^a)^: 2‐bromoethanol: base (molar ratio)	Reflux time [h]	Degree of substitution [%]
0.02: 0.02: 0.02	24	59.2
0.02: 0.03: 0.03	24	72.5
0.02: 0.05: 0.05	24	77.6
0.02: 0.06: 0.06	24	84.3
0.02: 0.06: 0.06	48	97.1

^a)^ Moles of L‐PEI taken as per repeat unit.

In order to minimize the toxic effects seen with L‐PEI, a high degree of substitution was desirable. Thus, reactions were conducted under reflux for 48 h using mole ratios of 0.02: 0.06: 0.06 (L‐PEI: 2‐bromoethanol: base). The 97% degree of substitution P2HEEI was used in all subsequent experiments.

**Figure 3:**
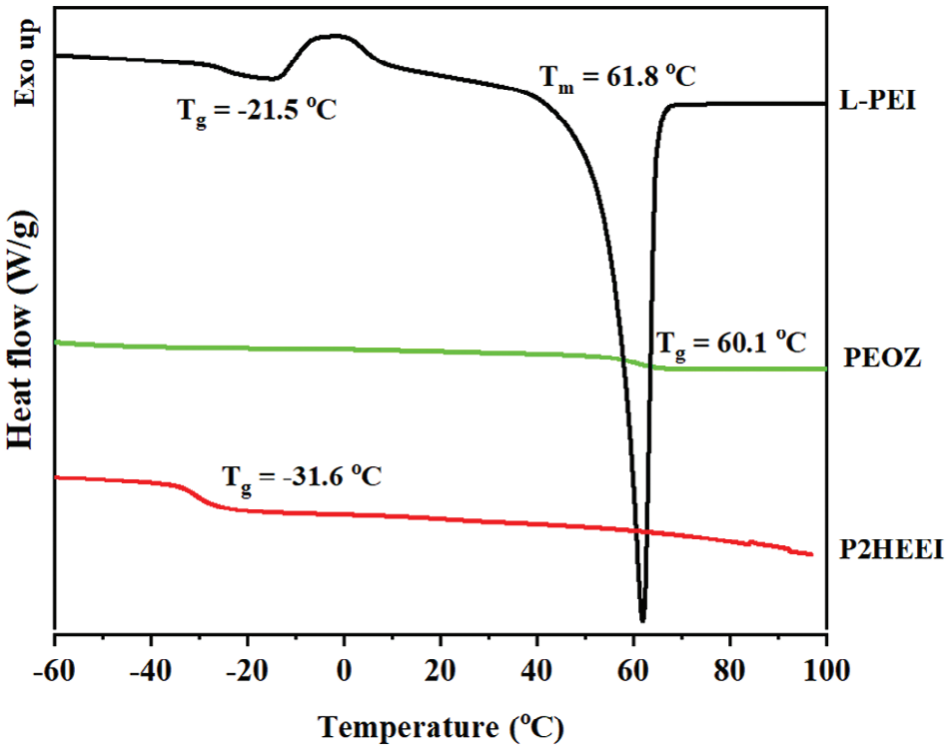
DSC thermograms of PEOZ, LPEI and P2HEEI.

PEOZ, L‐PEI, and P2HEEI were characterized by DSC and TGA. The glass transition temperatures (T_g_) of PEOZ, L‐PEI, and P2HEEI were 60.1, −21.5, and −31.6 °C, respectively (**Figure** **3**). Moreover, the DSC thermogram of L‐PEI indicated a melting point of 61.8 °C, which is consistent with previous studies.^[^ ^30^, ^36^ ^]^ P2HEEI, in contrast to L‐PEI, exhibited essentially amorphous behavior. The high chain flexibility of P2HEEI and very low value of T_g_ is consistent with the properties of some other water‐soluble polymers that have hydroxyl pendant groups, for example, poly(2‐hydroxyethyl vinyl ether), reported to have a T_g_ < −30 °C^[^ ^37^ ^]^ or poly(3‐hydroxypropyl ethyleneimine) with a T_g_ = −38.6 °C.^[^ ^15^ ^]^ The change from semi‐crystalline L‐PEI to amorphous P2HEEI was confirmed by X‐ray diffractometry (Figure S3, Supporting Information).

TGA was used to characterize the thermal stability of P2HEEI in comparison with PEOZ and L‐PEI (Figure S4, Supporting Information). Two distinct stages thermal decomposition stages are observed for the weight loss of PEOZ, L‐PEI, and P2HEEI. The first weight loss is attributed to free and physically‐bound water between 30 and 150 °C; the content of bound water was ≈2.0% for PEOZ, 4.2% for L‐PEI, and 5.0% for P2HEEI. The second loss of samples weight relates to thermal degradation of the polymers. The onset of decomposition was observed at 390 °C for PEOZ, 380 °C for L‐PEI, and 250 °C for P2HEEI, demonstrating that the new derivative is less thermally stable compared to its parent polymers.

**Figure 4:**
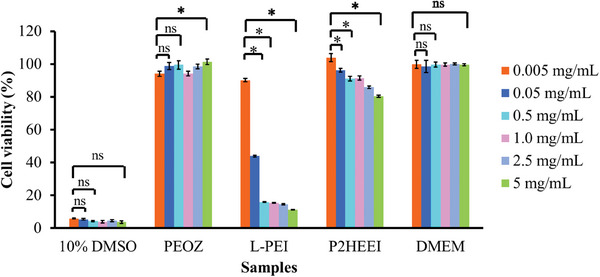
Human dermal fibroblasts viability in the presence of 10% DMSO (positive control), PEOZ, LPEI, P2HEEI, and DMEM (negative control) was assessed using MTT assay. Statistically significant differences are given as: ^*^ – *p* < 0.05; ns – no significance.

As this is the first report of the synthesis of P2HEEI with a high degree of hydroxyethylation, it was of interest to study its biocompatibility in comparison with PEOZ and L‐PEI. This was studied using an MTT assay with human dermal fibroblast cells (**Figure** **4**). P2HEEI was found to have relatively good biocompatibility and low toxicity with the fibroblasts >80% viable even when dosed at 5000 µg mL^−1^. On the contrary, L‐PEI was highly cytotoxic at concentrations ranging from 50 to 5000 µg mL^−1^, with less than 50% cell viability, in good agreement with the literature.^[^ ^15^, ^30^, ^32^ ^]^ PEOZ exhibited excellent biocompatibility, and did not cause any substantial levels of cell death across the broad range of concentrations (5–5000 µg mL^−1^), also in good agreement with the literature.^[^ ^38^ ^]^ In comparison with our earlier reports using a different modification to L‐PEI,^[^ ^15^ ^]^ cell viability of all concentrations of poly(2‐hydroxyethyl ethyleneimine) (P2HEEI, 5–5000 µg mL^−1^) was higher than for poly(3‐hydroxypropyl ethyleneimine) or P3HPEI. This suggests that the marginally longer alkyl chain in P3HPEI increased toxicity of modified L‐PEI in human dermal skin fibroblast cells, though both modified polymers showed low toxicity in human dermal skin fibroblast cells (and both significantly lower than for L‐PEI).

### 2.2 Preparation and Evaluation of Films

The low glass transition temperature (−31.6 °C) of P2HEEI is of interest for use in polymeric blends with other more rigid polymers where it may act as a plasticizer to improve the mechanical characteristics of the resulting blends. Here, the films formed from chitosan and P2HEEI were produced by casting from aqueous solutions with subsequent drying. Figure S5 (Supporting Information) and **Table** **2** show the FTIR spectra of films based on pure CHI, CHI/P2HEEI blends and pure P2HEEI. CHI displayed a broad peak above 3247 cm⁻¹ (OH and NH stretching), along with amide I (1625 cm⁻¹) and amide II (1514 cm⁻¹) bands. A peak at 1376 cm⁻¹ indicated incomplete deacetylation of chitosan,^[^ ^14^ ^]^ while the 1250 cm⁻¹ band corresponded to amino groups.^[^ ^13^ ^]^ The spectra of chitosan correlate with other reports.^[^ ^15^, ^39^, ^40^ ^]^ For P2HEEI, a broad band at 3314 cm⁻¹ indicated ‐OH stretching and bound water. The spectra of the CHI/P2HEEI blends showed substantial changes in the hydroxyl region, indicating a restructuring of the hydroxyl group associations. In the spectra of the blends with different composition, this band shifted toward higher wavenumbers (3247 to 3285 cm^−1^) as the content of P2HEEI increased from 0 to 80% w/v. This observation suggests that a significant portion of the hydroxyl and amine groups in chitosan are hydrogen‐bonded to hydroxyl groups in P2HEEI or that interactions between the polymers and water occur.

**Table 2:** FTIR absorption bands in CHI/P2HEEI blends and their assignment.

FTIR absorption of blends [cm^−1^]						Assignment
100:0	80:20	60:40	40:60	20:80	0:100	
3247	3259	3266	3270	3285	3314	OH‐ and NH‐stretching
2917	2923	2920	2912	2918	2940	CH‐stretching
2878	2883	2891	2880	2832	2818	CH‐stretching
1625^1^^,^^2^	1630^1,^ ^2^	1633^1,^ ^2^	1638^1,^ ^2^	1639^1,^ ^2^	16 48^2^	C═O stretching (amide I),^1^ water region^2^
1514^3^^,^^4^	1514^3,^ ^4^	1516^3,^ ^4^	1513^3,^ ^4^	1455^3,^4	1459^4^	NH‐bending (amide II),^3^ CH_2_ vibration^4^
1412	1414	1413	1412	1422	1424	CH‐ and OH vibration
1376^5^^,^^6^	1377^5,^ ^6^	1376^5,^ ^6^	1376^5,^ ^6^	1370^5,^ ^6^	1361^6^	Acetamide groups,^5^ CH‐vibration^6^
1311	1317	1311	1316	1306	–	CN‐ stretching (amide III)
1152	1151	1152	1152	1150	–	Anti‐symmetric stretching of the C─O─C bridge
1060^7^^,^^8^	10 63^7,^ ^8^	1061^7,^ ^8^	1062^7,^ ^8^	1028^7,^ ^8^	1029^8^	Skeletal vibration involving the C─O stretching,^7^ C─C stretching^8^

**Figure 5:**
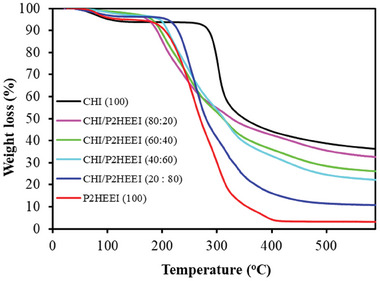
TGA thermograms of CHI, CHI/P2HEEI blends, and P2HEEI.

The thermal properties of CHI, CHI/P2HEEI, and P2HEEI were studied using TGA as shown in **Figure** **5**. Initially, CHI loses free‐ and physically‐bound water between 30 to 150 °C; ≈6% of the CHI film was bound water. CHI begins to thermally decompose between 250 and 400 °C, with the maximum degradation rate observed at 320 °C, resulting in 58.3% weight loss. Decomposition of chitosan results from degradation of its macromolecules and pyranose rings through dehydration and deamination and finally a ring‐opening reaction.^[^ ^14^ ^]^ There are also two distinct stages for weight loss from P2HEEI including free and physically‐bound water between 30 and 150 °C and then thermal decomposition, which begins at 250 °C. This demonstrates that the new polymer blend has lower thermal stability than the individual components. The decomposition profiles of CHI/P2HEEI blends are essentially the sum of their components with initial loss of water followed by degradation of P2HEEI and then degradation of chitosan.

**Figure 6:**
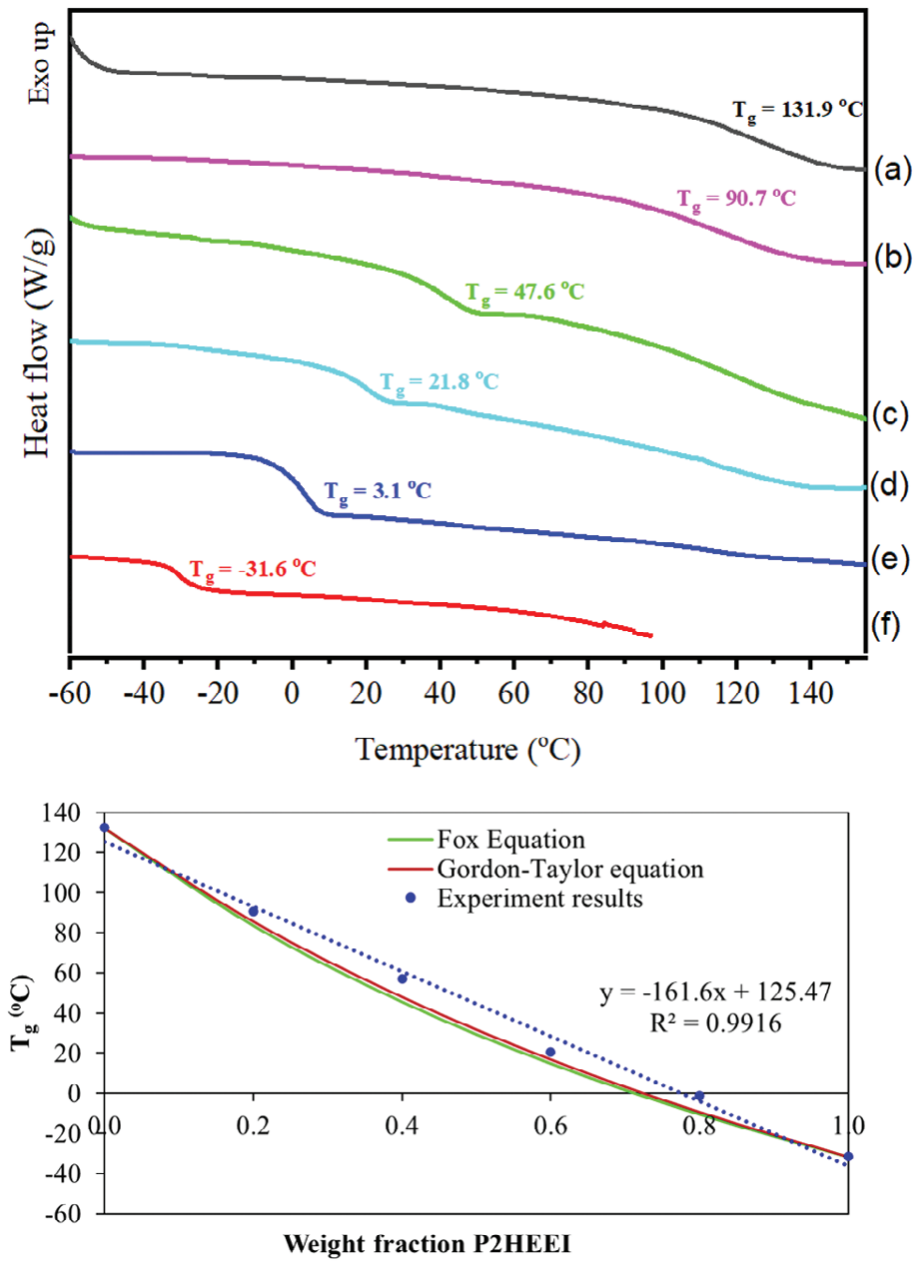
DSC thermograms of CHI a), their blends b–e) and P2HEEI f). Content of P2HEEI in the blends: 20 (b), 40 (c), 60 (d), and 80% (e) (6a) and correlation between the weight fraction of P2HEEI in P2HEEI‐chitosan blends and Tg of experimental result compared with theoretical results (6b).

The miscibility of the polymers in the blends was investigated using DSC. It is well established that a single, intermediate T_g_ strongly indicates the miscibility of polymer blend components. **Figure** **6**a does indeed show a single glass transition in the blends, which is dependent on the polymer composition; T_g_s of CHI/P2HEEI blends were between the T_g_ values of individual P2HEEI (−31.6 °C) and chitosan (131.9 °C). P2HEEI appears to act as a plasticizer for these blends. It is well‐known that traces of water can plasticize water‐soluble polymers, significantly lowering the T_g_.^[^ ^37^, ^41^ ^]^ P2HEEI is structurally similar to poly(2‐hydroxyethyl vinyl ether), which has a T_g_ < −30 °C.^[^ ^37^ ^]^ Blending poly(acrylic acid) with poly(2‐hydroxyethyl vinyl ether) also resulted in a substantial reduction of T_g_ of the films.^[^ ^37^ ^]^

Figure 6b shows the relationship between weight fraction of P2HEEI and T_g_ for the experimental results and those calculated theoretically. The T_g_ of miscible blends can be predicted by using Fox^[^ ^42^ ^]^ and Gordon–Taylor^[^ ^43^ ^]^ expressed by Equations (1) and (2):

(1) $$\frac{1}{\;T_{g}}=\frac{W_{\mathrm{CHI}}}{T_{g,\mathrm{CHI}}}+\frac{W_{P2\mathrm{HEEI}}}{T_{g,P2\mathrm{HEI}}}\left(\mathrm{Fox}\;\mathrm{equation}\right)$$

(2) $$\begin{array}{cc} T_{g} & =\frac{W_{\mathrm{CHI}}T_{g,\mathrm{CHI}}+kW_{P2\mathrm{HEEI}}T_{g,P2\mathrm{HEEI}}}{W_{\mathrm{CHI}}+kW_{P2\mathrm{HEEI}}}\\ & \;\times (\mathrm{Gordon}-\mathrm{Taylor}\;\mathrm{equation}) \end{array}$$

where W_CHI_ and W_P2HEEI_ are the weight fractions of chitosan and P2HEEI, respectively; and T_g,CHI_ and T_g,P2HEEI_ are the glass transition temperatures of chitosan and P2HEEI, respectively; k is the ratio of heat capacity change of P2HEEI over chitosan [k = ΔC_p2_/ C_p1_)].^[^ ^44^ ^]^

The T_g_ of the blends tended to decrease as the P2HEEI concentration increased, and their values were in good agreement with those calculated from theory. The correlation curve between the weight fraction of P2HEEI and the T_g_ of CHI/P2HEEIl blends obtained from experimental data was above the theoretical plots from the Fox and Gordon‐Taylor equations, demonstrating greater interaction between the components than expected from simple mixing which aids compatibility.^[^ ^45^ ^]^ These findings suggested that a miscible phase was formed at the molecular level in these blends, and that intermolecular hydrogen bonds are likely to form between the ‐OH and ‐NH_2_ groups of chitosan and the ‐OH groups of P2HEEI.

**Figure 7:**
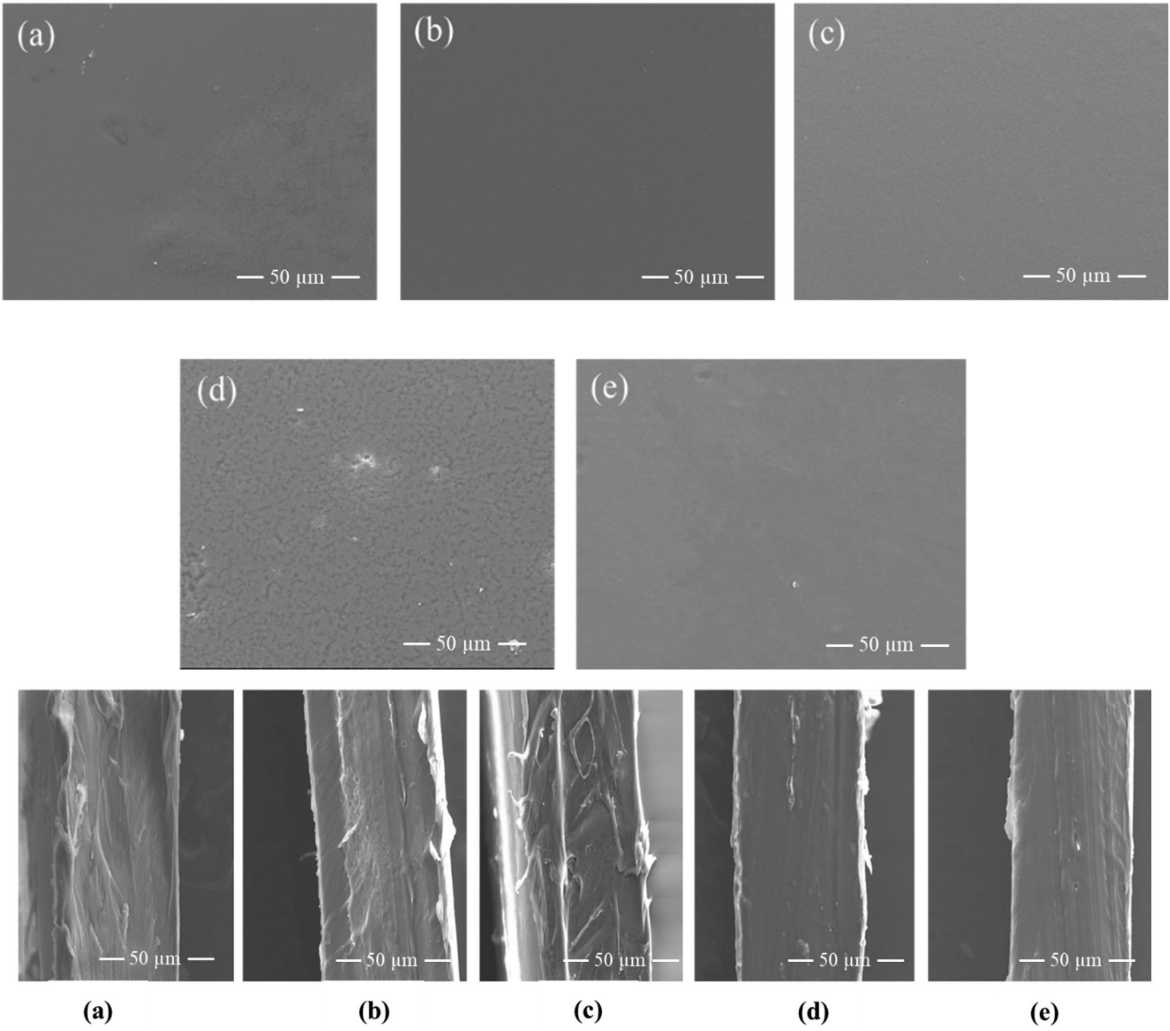
SEM images of film surfaces A) and cross‐sections B) of CHI (a) and their blends with P2HEEI (b–e). Content of P2HEEI in the blends: 20 (b), 40 (c), 60 (d) and 80% (e).

The microstructure of the polymer films viewed in cross‐section and from the surface was imaged using SEM. The sample cross‐sections showed that the films appear uniform with no signs of phase separation (**Figure** **7**). A similar lack of phase separation was observed on the film surfaces. The SEM results provided further evidence for miscibility in CHI‐P2HEEI blends. The SEM results correlated well with fluorescent microscopy evaluation of the films prepared using fluorescently‐labelled chitosan (Figure S7, Supporting Information); the fluorescence images also show no evidence of phase separation in these blends.

X‐ray diffraction patterns recorded for CHI/P2HEEI films indicated possible interactions between CHI and P2HEEI (Figure S8, Supporting Information). CHI is a semi‐crystalline polysaccharide with crystalline domains that typically show several diffraction peaks.^[^ ^46^ ^]^ The diffraction pattern of pure chitosan film recorded in the current study has a relatively low signal‐to‐noise ratio, which is possibly related to the thinner films used here compared to Abilova et al.^[^ ^14^ ^]^ However, the diffraction peak at 12.9 ° typical for CHI is still clearly visible. The absence of crystalline characteristics in the diffraction pattern of pure P2HEEI and the presence of a broad amorphous halo indicate that this polymer was amorphous. The X‐ray diffraction spectra of CHI/P2HEEI films similarly display a large halo, which is typical for predominately amorphous polymers. In addition, when the amount of P2HEEI in the films increased, the sharper chitosan diffraction peaks disappeared, potentially due to simple dilution effects or, alternatively, molecular mixing reducing the intermolecular hydrogen bonding of the chitosan crystalline domains.

**Figure 8:**
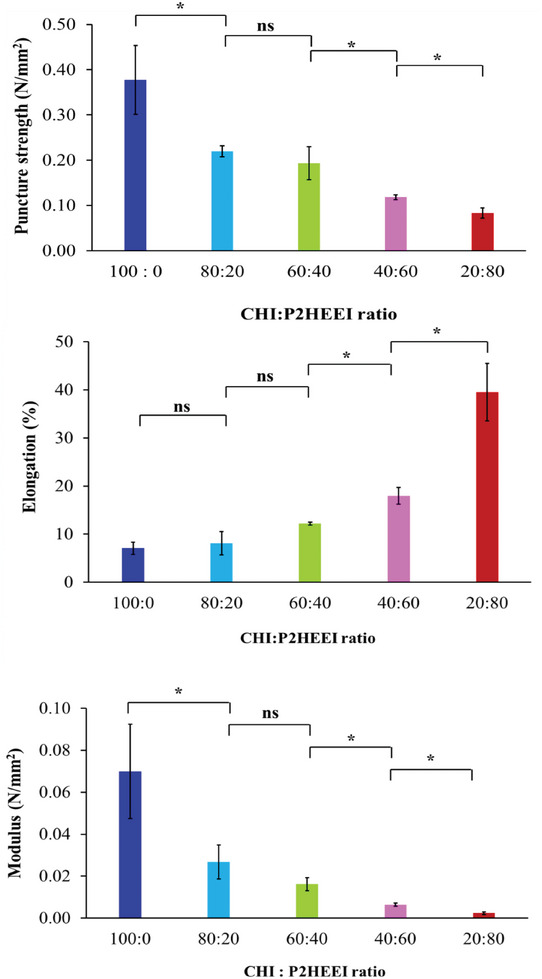
Mechanical properties of CHI and their blends with P2HEEI. Statistically significant differences are given as: ^*^ – *p* < 0.05; ns – no significance.

**Figure** **8** illustrates the mechanical characteristics of the films prepared from pure CHI and its blends with P2HEEI. The results indicated that pure chitosan films had a greater puncture strength (0.38 N mm^−2^) but lower elongation (5.6%) than the blends. When the P2HEEI content increased, the puncture strength of CHI/P2HEEI films decreased significantly (*p* < 0.05), whereas the flexibility of CHI/P2HEEI films tended to increase in terms of elongation (*p* < 0.05). The modulus at puncture was calculated from the correlation between puncture strength over elongation, previously been used to calculate the rigidity or stiffness of materials. Because of the high strength and low percentage of elongation, the modulus was high. This study found that increasing the P2HEEI content in CHI/P2HEEI films resulted in a decrease in modulus at puncture, indicating that increasing the P2HEEI content results in more elastic materials. As shown above, P2HEEI has a relatively low T_g_ (−31.6 °C), and therefore acts as a plasticizer.^[^ ^37^ ^]^ Plasticizers are usually small or oligomeric molecules that insert between polymer chains, breaking hydrogen bonds and spacing the chains apart to improve flexibility.^[^ ^42^ ^]^ As a result, increasing the P2HEEI concentration in CHI/P2HEEI films probably reduced intermolecular hydrogen interactions between CHI macromolecules, resulting in improved chain mobility and film flexibility.^[^ ^47^ ^]^ This result correlates with DSC data, as the higher elasticity of CHI/P2HEEI films with an increasing P2HEEI content is associated with a decrease in T_g_ of CHI/P2HEEI films.

### 2.3 Chitosan and Poly(2‐hydroxyethyl ethyleneimine) Films for Buccal Delivery of Haloperidol: In Vitro Drug Release and Ex Vivo Mucoadhesion Studies

Haloperidol (HP) was selected as a model poorly water‐soluble drug for buccal drug delivery. HP is an antipsychotic drug and its use is often associated with extrapyramidal syndrome side effects manifested as involuntary body movements that cannot be easily controlled.^[^ ^48^ ^]^ It is usually formulated as solutions for oral administration or injections, and as tablets.^[^ ^35^ ^]^ The typical daily oral dose of haloperidol ranges from 0.5 to 30 mg.^[^ ^49^ ^]^ Additionally, it is a BCS class 2 drug, defined by low solubility and high permeability, with limited oral bioavailability at 59%.^[^ ^49^, ^50^ ^]^ Hence, developing a haloperidol formulation for buccal administration is of interest and so drug‐loaded films based on blends of CHI and P2HEEI were produced for this purpose.

**Figure 9:**
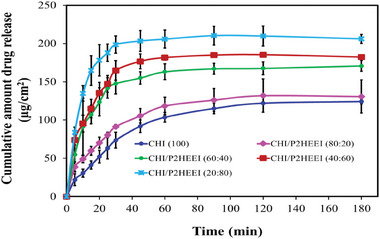
Cumulative haloperidol release per unit area of drug loaded films of CHI and blends with P2HEEI.

Drug release from these films was studied using a Franz diffusion cell with 20% PEG 400‐PBS as receiver medium (pH 7.4 at 37 °C). **Figure** **9** illustrates the cumulative release profiles from HP‐loaded CHI/P2HEEI films prepared at different polymer ratios. Four commonly applied models were used to assess the kinetics of drug release from the films; zero order, first order, Higuchi and Korsmeyer‐Peppas models (Figure S9 and Table S2, Supporting Information). No single model fitted the data well, reflecting the complex processes involved including diffusion of the drug through the films as they interact with the receiver fluid, and erosion and dissolution of the two components in the film.

Drug release varied significantly (*p* < 0.05) as the CHI‐P2HEEI ratio changed in the films. Incorporating the drug into pure CHI films gave the slowest haloperidol release (no more than≈125 µg cm^−2^) within 180 min. The addition of P2HEEI to CHI in the films from 0 to 80% (w/v) resulted in an increase in the drug release from ≈135 to ≈207 µg cm^−2^. This faster release of haloperidol from the films plasticized with P2HEEI is likely related to their increased elasticity that facilitates the diffusion of drug (and solvent) molecules. In addition, the glass transition temperature (Tg) of a polymer is a crucial factor in drug delivery systems, as it directly affects the drug release profile. Above Tg, the polymer chains become more flexible, promoting drug release. In contrast, a higher Tg makes the polymer more rigid, limiting drug release due to reduced molecular mobility. Furthermore, an increased P2HEEI content in CHI/P2HEEI blend films enhances water diffusion, swelling, and erosion, leading to the relaxation of polymer chains due to their lower Tg. This results in rapid drug release, followed by film disintegration. Additionally, higher P2HEEI content in the films contributes to increased drug release and higher drug loading, facilitating dose optimization.

**Figure 10:**
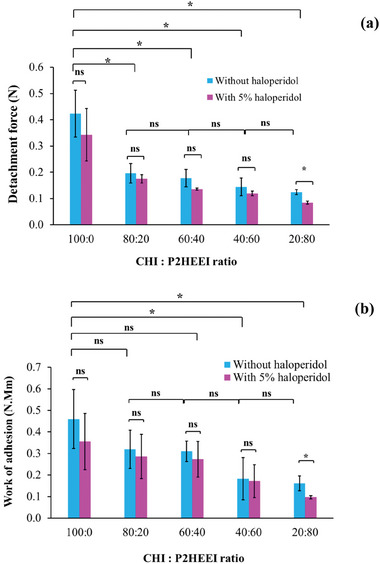
Detachment force a) and work of adhesion b) of CHI/P2HEEI films with and without haloperidol evaluated in sheep buccal mucosa as a function of P2HEEI content in the blends. Statistically significant differences are given as: ^*^ – *p* < 0.05; ns – no significance.

For buccal drug delivery, suitable film mucoadhesion allows longer residence on the buccal mucosa to ensure high drug concentrations at the site of administration^[^ ^51^ ^]^ whilst providing a suitable “mouth feel” for the delivery device. Tensile methods are commonly used to examine the mucoadhesive characteristics of various formulations (films, tablets and granules).^[^ ^52^ ^]^ In this work, sheep buccal tissues were used as a substrate for mucoadhesion experiments. CHI and CHI/P2HEEI films, with and without haloperidol, were placed on, and then detached from, the buccal tissue, and the maximum detachment force (F_adh_) and total work of adhesion (W_adh_) were determined (**Figure** **10**). The results clearly indicated that films based on chitosan alone exhibited greatest mucoadhesive properties with the highest F_adh_ (0.42 ± 0.09 N) and W_adh_ (0.45 ± 0.13 N⋅mm). It was expected as chitosan is known for its excellent mucoadhesive ability.^[^ ^53^ ^]^ As the amount of P2HEEI in the films increased, the values for detachment force and total effort of adhesion decreased progressively. The F_adh_ declined significantly (*P* < 0.05) from 0.42 to 0.12 N and the W_adh_ fell significantly (*P* < 0.05) from 0.45 to 0.16 N⋅mm when P2HEEI was added to chitosan at 80% (w/w). This trend was also consistent with our previous research on the mucoadhesive properties of chitosan blends containing hydroxyethylcellulose (HEC) on porcine buccal mucosa,^[^ ^13^ ^]^ where mucoadhesion decreased with increasing HEC content. Abilova and co‐workers^[^ ^54^ ^]^ reported the development of mucoadhesive films based on chitosan and poly(2‐ethyl‐2‐oxazoline) and showed increasing poly(2‐ethyl‐2‐oxazoline) content in the films reduced their adhesive properties.^[^ ^54^ ^]^ In our studies, mucoadhesion of 5% haloperidol‐loaded CHI and CHI/P2HEEI films was lower compared to the films without haloperidol, probably due to the inability of small drug molecules to contribute to mucosal adhesion. Similar observation was reported previously in CHI/PEOZ films containing ciprofloxacin.^[^ ^54^ ^]^

## 3 Conclusion and Outlook

Poly(2‐hydroxyethyl ethyleneimine) was synthesized successfully via reaction of L‐PEI with 2‐bromoethanol. Increasing the molar ratio of 2‐bromoethanol to L‐PEI and increasing the reflux time resulted in higher degrees of substitution of P2HEEI and the highest DS systems (97.1%) was selected to potentially reduce toxicity of L‐PEI. P2HEEI displayed excellent solubility in water, low toxicity, and had a low glass transition temperature (−31.6 °C). P2HEEI was then blended with chitosan providing flexible films. Miscibility and physicochemical properties of these films were investigated and indicated that the polymers in the blends were miscible. Chitosan and P2HEEI blends significantly improved the elasticity and mechanical strength of the films compared to chitosan or P2HEEI alone. An increase in the amount of P2HEEI in the blends resulted in a more rapid and greater cumulative release of haloperidol from the films. Using an ex vivo mucoadhesion study with sheep buccal tissue, films based on pure chitosan and its blends with poly(2‐hydroxyethyl ethyleneimine) demonstrated mucoadhesive properties; however, this property decreased significantly when the amount of P2HEEI in the films was increased. Therefore, blending chitosan with P2HEEI offers a simple way of modulating the mucoadhesive properties as well as drug release profiles and these formulations have the potential to be used as elastic films for the buccal drug delivery.

## 4 Experimental Section

### Materials

Chitosan (CHI, with the molecular weight of ≈310–375 kDa with the degree of deacetylation of 75%–85%), poly(2‐ethyl‐2‐oxazoline) (PEOZ, with a molecular weight of ≈50 kDa and a dispersity (Ɖ) of 3–4; experimental gel permeation chromatography analysis determined its weight‐average molecular weight to be 24.8 kDa with a dispersity of 1.76^[^ ^55^ ^]^), 2‐bromoethanol, 37% HCl solution, fluorescein isothiocyanate (FITC) and haloperidol were acquired from Merck (Gillingham, UK). Phosphate‐buffered saline (PBS) tablets and NaOH were purchased from Fisher Chemicals (Fisher Scientific, UK). The freshly excised sheep upper and lower lips were sourced from PC Turner Abattoir (Farnborough, Hampshire, UK).

### Synthesis of Linear Polyethyleneimine

L‐PEI was synthesized from poly(2‐ethyl‐2‐oxazoline) (PEOZ) using the protocol reported by our group previously.^[^ ^35^, ^56^ ^]^ In brief, 10 g of PEOZ was dissolved in 100 mL of 18.0% (w/w) HCl and refluxed at 100 °C for 14 h. The resulting L‐PEI solution in HCl was then diluted with deionized water (500 mL). Aqueous 4M solution of NaOH was added to the suspension until the L‐PEI precipitated at a pH of 10–11.^[^ ^57^ ^]^ The precipitate was filtered, rinsed with deionized water, and re‐precipitated two times before vacuum drying to obtain L‐PEI as a white powder, yielding 3.8 g (89%).

### Synthesis of Poly(2‐hydroxyethyl ethyleneimine)

P2HEEI was synthesized by nucleophilic substitution, using a protocol adapted from our previous study.^[^ ^56^ ^]^ Briefly, L‐PEI (1.0 g) was dissolved in 60 mL ethanol in a three‐necked round bottom flask, and different quantities of 2‐bromoethanol (0.02, 0.03, 0.05 and 0.06 moles) were added. Then, different quantities of potassium carbonate (0.02, 0.03, 0.05, and 0.06 moles) were added to this solution. The amounts of 2‐bromoethanol and potassium carbonate used are shown in **Table** **3**. The reaction mixture was refluxed at 78 °C for 24–48 h. Upon completion, it was centrifuged, and the supernatant was collected and concentrated using a rotary evaporator at 40 °C (280 rpm). The resulting mixture was diluted with deionized water and purified by dialysis against deionized water using a cellulose membrane (MWCO 3.5 kDa). P2HEEI was obtained as a dry residue through freeze‐drying.^1^H‐NMR and FTIR spectroscopies were used to confirm successful conversion of L‐PEI to P2HEEI.

**Table 3:** Amounts of L‐PEI, 2‐bromoethanol and potassium carbonate (base) used in the synthesis of poly(2‐hydroxyethyl ethyleneimine).

L‐PEI: 2‐bromoethanol: base (mole ratios)	L‐PEI [g]	2‐bromoethanol [mL]	Potassium carbonate [g]
0.02: 0.02: 0.02	1.0	1.7	3.2
0.02: 0.03: 0.03	1.0	2.5	4.8
0.02: 0.05: 0.05	1.0	3.3	6.4
0.02: 0.06: 0.06	1.0	4.1	8.1

### Preparation of Films

Films were prepared from chitosan (CHI) and its mixtures with P2HEEI by casting polymer solutions with subsequent evaporation of solvent. First, 1% w/v solutions of CHI and P2HEEI were prepared; the CHI solution (pH 2.0) was prepared in 0.1 M HCl by continuous stirring for 24 h, while P2HEEI solutions (pH 6.8) were made in deionized water and stirred for 1 h. These solutions were also mixed at different volume ratios, termed CHI (100: 0), CHI/P2HEEI: (80:20), (60:40), (40:60) and (20:80). Subsequently, all CHI/P2HEEI mixtures were stirred for 3 h. The pH of the mixtures was 3.0–4.0. 45 mL of each polymer mixture was poured into Petri dishes (90 mm diameter) and dried at 30 ± 2 °C in an oven.

### Preparation of Films Loaded with Haloperidol

A 5 mg/mL haloperidol solution was prepared in ethanol. Then, 1 mL of haloperidol solution was mixed with 9 mL of CHI and CHI/P2HEEI solutions for 2 h to generate the final 5 mg haloperidol film loading. 1% CHI solution and 1% CHI/P2HEEI solution were prepared as above but here, 10 mL of solution was decanted into Petri dishes (35 mm in diameter) before drying.

### Characterization of Polymer —^1^H‐Nuclear Magnetic Resonance Spectroscopy (^1^H‐NMR)

^1^H NMR spectra were recorded from 20 mg mL^−1^ PEOZ and L‐PEI prepared in methanol‐d_4_ and 20 mg mL^−1^ P2HEEI prepared in D_2_O using a 400 MHz Ultrashield PlusM B‐ACS 60 spectrometer (Bruker, UK). The degree of substitution (DS) of P2HEEI was calculated using the following Equation (3):

(3) $$\%\;\mathrm{DS}\;=\frac{\int \mathrm{Peak}\;b/n_{b\;}}{\int \mathrm{Peak}\;a/n_{a}}x\;100$$

where Peak a is the integral of CH_2_CH_2_ signal on the backbone, Peak b is the integral of CH_2_ side‐group signal, n_a_ is the number of protons of CH_2_CH_2_ on the backbone and n_b_ is the number of protons in CH_2_ on the side‐group.

### Characterization of Polymer and Films—Fourier Transformed Infrared Spectroscopy (FTIR)

FTIR spectra were recorded for dry polymer samples and the films formed on a Nicolet iS5‐iD5 ATR FT‐IR spectrometer (Thermo Scientific, UK). All samples were scanned from 4000 to 400 cm^−1^ at a resolution of 4 cm^−1^ taking 64 scans.

### Characterization of Polymer and Films—Differential Scanning Calorimetry (DSC)

DSC characterization of polymer and film samples was conducted using a Q100 DSC (TA Instruments, Germany) in a nitrogen atmosphere. The DSC thermograms were recorded for the polymer samples (≈3–5 mg) in pierced T_zero_ aluminum pans with a heating/cooling rate of 10 °C min^−1^ (−70 to 180 °C). Glass transition temperature (T_g_) values were obtained from the second heating cycle.

### Characterization of Polymer and Films—Thermogravimetric Analysis (TGA)

TGA of polymer and film samples was performed using a Q50 TGA analyzer (TA Instruments, UK) by heating from 20 to 600 °C at 10 °C min^−1^ in a nitrogen atmosphere. The films were dried in a vacuum oven (as described earlier) and then stored in a desiccator over dry silica gel for 3 days before TGA analysis.

### Characterization of Polymer and Films—X‐Ray Diffractometry (XRD)

Approximately 20 mg of dry polymers or 2×2 cm^2^ films were placed on a silica slide for analysis using a Bruker D8 ADVANCE PXRD system, which features a LynxEye detector and monochromatic Cu Kα1 radiation (λ = 1.5406 Å). During the analysis, the samples were rotated at 30 rpm. Data collection occurred over a 2θ angular range of 5–60° for one hour, utilizing a step size of 0.05° (2θ) and a count time of 1.2 s per step.

### Cytotoxicity of Polymers

MTT assay was used to evaluate the cytotoxicity of polymers. L‐PEI was dissolved in 95% ethanol and then diluted with Dulbecco's modified eagle medium (DMEM) to prepare solutions with the polymer concentrations between 5 and 5000 µg mL^−^. PEOZ and P2HEEI were dissolved directly in DMEM and then diluted to form solutions at the same concentrations. Human dermal fibroblasts (ATCC CRL‐2522) were seeded at 1 × 10^5^ cells mL^−1^ per well in a 96 well plate and allowed to attach overnight before incubation with the respective polymer solutions at 5, 50, 500, 1000, 2500, and 5000 µg mL^−1^ for 24 h. 10% DMSO (v/v) in Dulbecco's modified eagle medium (DMEM) was used as a positive control and 10% fetal bovine serum in DMEM was the negative control. 100 µL of 3‐(4, 5‐dimethylthiazol‐2‐yl)‐2, 5‐diphenyl tetrazolium bromide solution (MTT) solution was then pipetted into each well and the plate incubated at 37 °C in a CO_2_ incubator for 3 h. The amount of formazan formed was determined by monitoring absorbance at 570 nm with a plate reader (Thermo Scientific™ Multiskan™ GO, Finland).

### Characterization of Films—Film Thickness

Film thickness was characterized with a digital micrometer (Mitutoyo, Japan). Multiple measurements were taken at different points of each film from which the mean ± standard deviation was calculated (Table S1, Supporting Information).

### Characterization of Films—Scanning Electron Microscopy (SEM)

SEM analysis was performed using a FEI Quanta 600 FEG Environmental Scanning Electron Microscope (FEI UK Ltd., UK) at an acceleration voltage of 20 kV. Images were captured from the fracture surfaces of the films, which were coated with a layer of gold using a diode sputter.

### Characterization of Films—Fluorescence Microscopy

Fluorescein isothiocyanate (FITC)‐labeled chitosan was synthesized following the protocol described by Cook et al.^[^ ^58^ ^]^ Initially, 1% w/v aqueous solutions of FITC‐labeled chitosan and P2HEEI were prepared by dissolving pre‐weighed amount of dry polymers at room temperature. FITC‐labeled chitosan solution was prepared in 0.1 m hydrochloric acid by stirring for 24 h prior to casting. P2HEEI solutions were prepared in deionized water and stirred for 1 h. These solutions were mixed at different volume ratios, termed FITC‐labeled chitosan (100:0), FITC‐labeled chitosan /P2HEEI: (80:20), (60:40), (40:60), and (20:80). Subsequently, FITC‐labeled chitosan /P2HEEI solutions were stirred for 3 h before 10 mL of each solution was poured into Petri dishes (30 mm in diameter) and dried at 30 °C in an oven for 3–5 days. The morphology of film samples was analyzed using a fluorescence microscope (CARY Eclipse, US).

### Characterization of Films—Mechanical Studies of Films

The mechanical characteristics of films, including puncture strength, elongation at puncture and modulus at puncture were evaluated using a TA.XT Plus Texture Analyzer (Stable Micro Systems Ltd., UK) in compression mode.^[^ ^14^, ^59^, ^60^ ^]^ Square samples measuring 30×30 mm were placed between two plates with a cylindrical hole of 10 mm in diameter (with a sample holder hole area of Ars = 78.57 mm^2^). A 5 mm stainless steel spherical ball probe (P/5S) was then used to compress the samples at a speed of 1.0 mm s^−1^. To prevent movement, the plates were stabilized with two pins. The test parameters were: pre‐test speed of 2.0 mm s^−1^, test speed of 1.0 mm s^−1^, post‐test speed of 10.0 mm s^−1^, target mode: distance; distance: 5 mm, trigger type: auto, and trigger force: 0.049 N. Puncture strength was calculated using the Equations (4) and (6) based on the punctured film samples:

(4) $$\mathrm{Puncture}\;\mathrm{strength}=\frac{F_{\max}}{Ar_{s}}$$

where F_max_ is the maximum applied force, Ar_s_ is the area of the sample holder hole, with Ar_s_ = πr^2^, where r is the radius of the hole.

(5) $$\mathrm{Elongation}\;\left(\%\right)=\left(\frac{\sqrt{r^{2}+d^{2}}-r}{r}\right)x100$$

where r is the radius of the film exposed in the cylindrical hole of the film holder and d represents the displacement of the probe from the point of contact to the point of puncture.

(6) $$\mathrm{Modulus}\;\mathrm{at}\;\mathrm{puncture}=\frac{\mathrm{Puncture}\;\mathrm{strength}}{\mathrm{Elongation}\;\left(\%\right)}$$

### Drug Release Studies

Release of haloperidol from the films was determined using Franz diffusion cells and a methodology adapted from Samanta et al.^[^ ^48^ ^]^ and Soradech et al.^[^ ^15^ ^]^ The receptor compartment was filled with 20 mL of 20% PEG 400 in PBS solution (pH 7.4)^[^ ^35^ ^]^ and stirred continuously at 600 rpm at 37 °C. 5% haloperidol loaded films were placed between the two compartments of the Franz cell. At specified time intervals, 1 mL aliquots were taken from the receptor compartment and replaced with 1 mL of fresh medium (20% PEG 400 in PBS solution). All release experiments were carried out for 180 min. The drug concentration in the aliquot was measured using spectrophotometry at 254 nm and calculated using a standard calibration curve with the drug concentration range of 5–50 µg mL^−1^. The procedure used for preparing the stock solution of haloperidol and standard curve was followed from our previous study.^[^ ^15^ ^]^ For each type of film, three replicate experiments were conducted.

### Ex Vivo Mucoadhesive Properties of Film with and Without Haloperidol

Sheep buccal mucosal tissues were used for evaluation of mucoadhesive properties. Adhesion of the polymeric films with and without haloperidol was determined using a TA XT plus Texture Analyzer (Stable Microsystems, UK) in a tensile mode, as in our previous studies^[^ ^54^ ^]^ and illustrated in Figure S10 (Supporting information). The films (1×1 cm) were attached to the texture analyzer probe using double‐sided adhesive tape. The mucosal tissue was secured onto the sample holder and moistened with 1 mL PBS solution. The films were then placed in contact with the mucosa, and a downward force of 0.1 N was applied for 60 s. Afterward, the probe was withdrawn from the mucosa at 1 mm s^−1^. For each type of films, five replicate experiments were performed.

### Statistical Analysis

The results are presented as mean values ± standard deviation, which were calculated as a result of 3 independent experiments. One‐way ANOVA and Student's t‐test were used for the analysis of the data to determine the extent of any differences between the results.

## Conflict of Interest

The authors declare no conflict of interest.

## Supporting information



Supporting Information

## Data Availability

The data that support the findings of this study are available in the supplementary material of this article.
