# Supplementary material for: Synthesis of Poly (2‐hydroxyethyl ethyleneimine) and Its Mucoadhesive Film Formulations When Blended with Chitosan for Buccal Delivery of Haloperidol
Source: Macromol Biosci. 2025 Mar 3;25(6):2400642. doi: 10.1002/mabi.202400642 (PMC12169502; doi:10.1002/mabi.202400642)
Supplement: Supplementary file 1 — Supporting Information [file MABI-25-2400642-s001.docx]

**Supporting Information**

**Synthesis of poly(2-hydroxyethyl ethyleneimine) and its mucoadhesive film formulations based on blends with chitosan for buccal delivery of haloperidol**

Sitthiphong Soradech ^1, 2^, Adrian C. Williams ^1^and Vitaliy V. Khutoryanskiy ^1*^

^1^ Reading School of Pharmacy, University of Reading, Whiteknights, Reading, RG6 6AX, UK

^2^ Expert Centre of Innovative Herbal Products, Thailand Institute of Scientific and Technological Research, Pathum Thani, 12120, Thailand.

***Corresponding author:**

Postal address: School of Pharmacy, University of Reading, Whiteknights, RG6 6AX, Reading, United Kingdom

E-mail address: [v.khutoryanskiy@reading.ac.uk](mailto:v.khutoryanskiy@reading.ac.uk)

Telephone: +44(0) 118 378 6119

Fax: +44(0) 118 378 4703


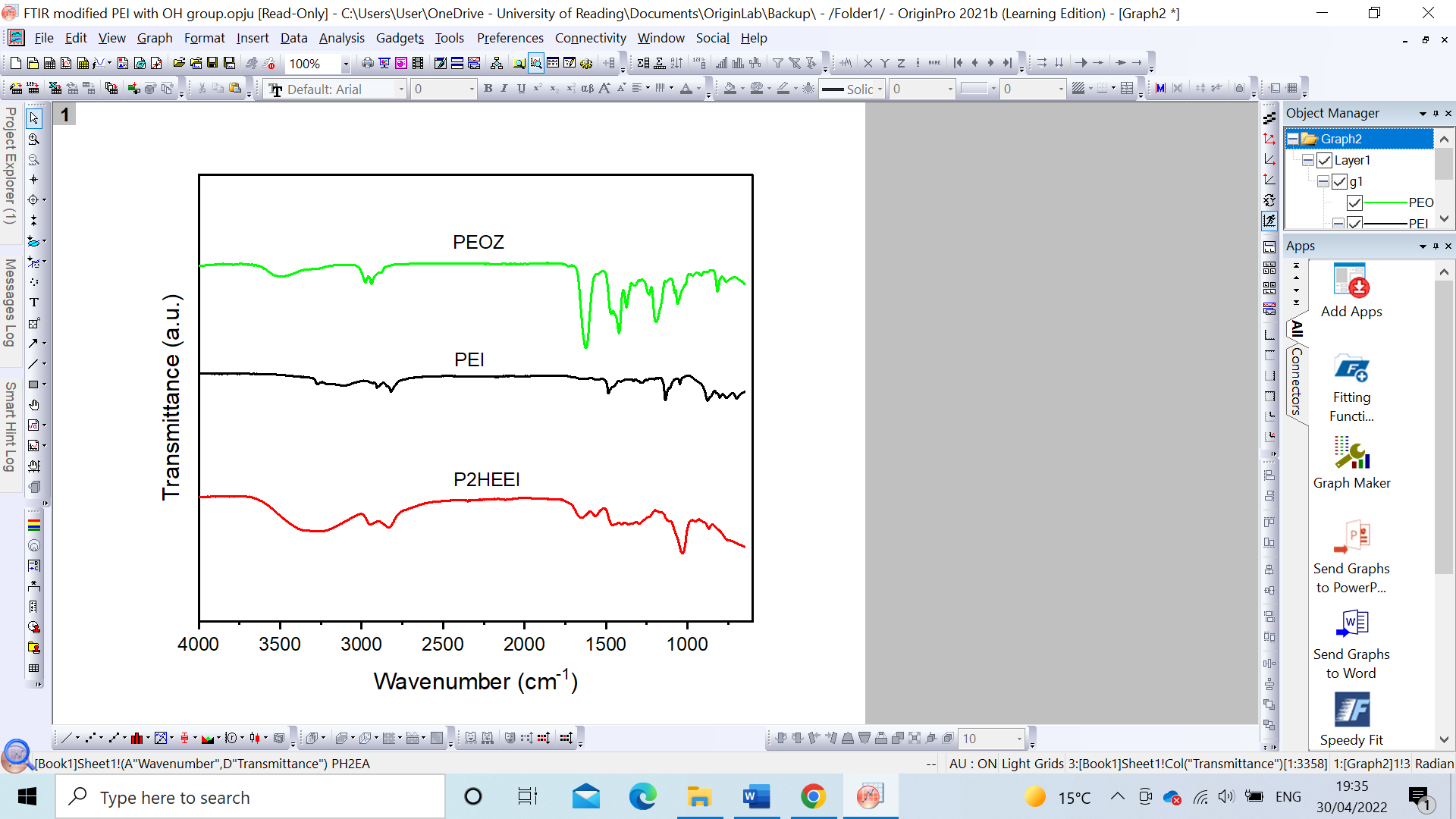


**Figure S1.**  FTIR spectra of PEOZ, LPEI, and P2HEEI.


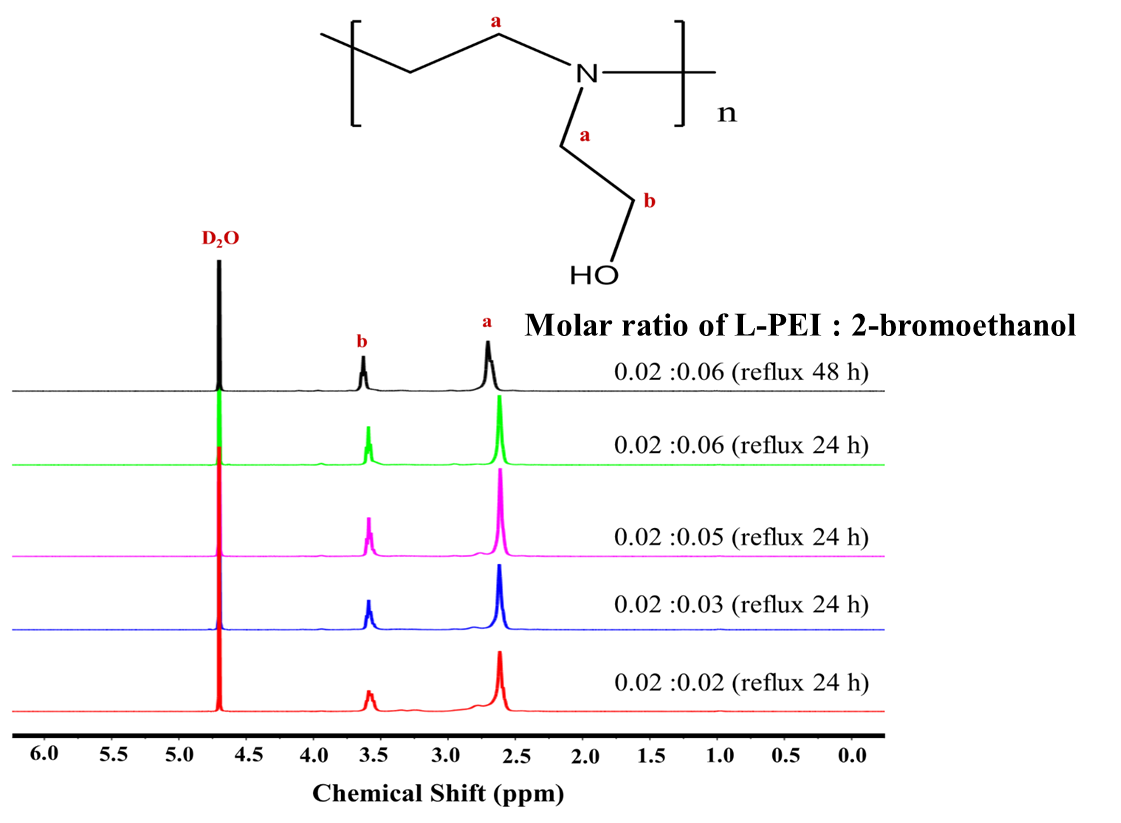


**Figure S2.** ^1^H NMR spectra of hydroxyethyl substituted linear polyethyleneimine prepared at different mole ratios of L-PEI:2-bromoethanol and reflux time.


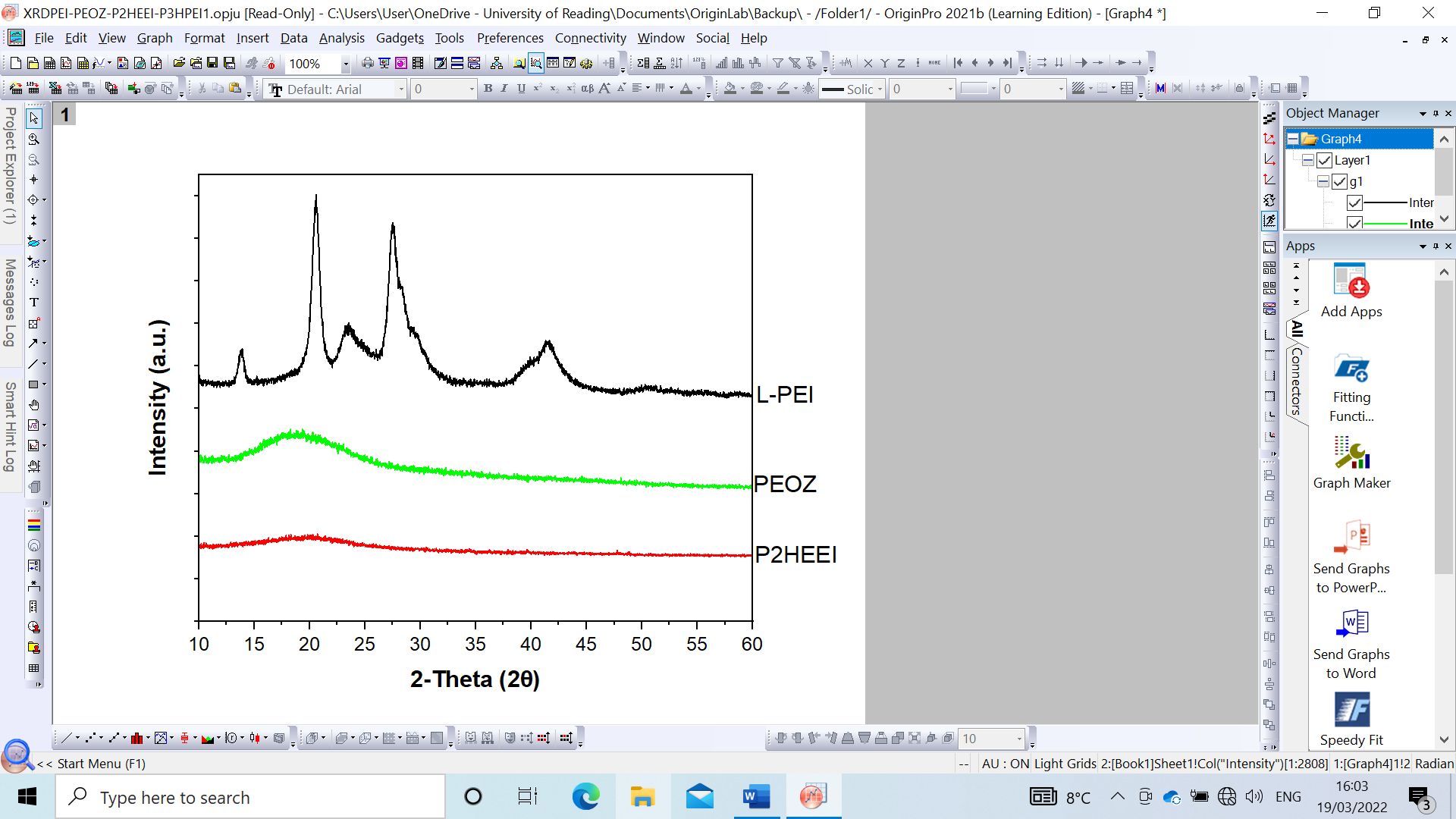


**Figure S3.** X-ray diffractograms of PEOZ, LPEI and P2HEEI.

**
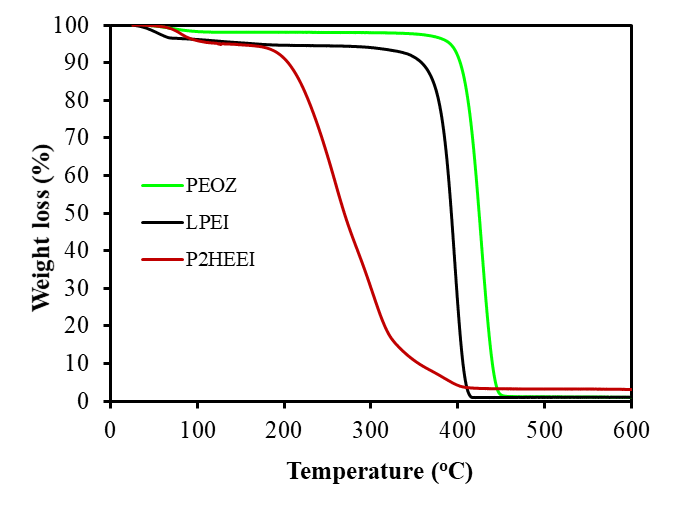
**

**Figure S4.**  TGA thermograms of PEOZ, LPEI and P2HEEI.


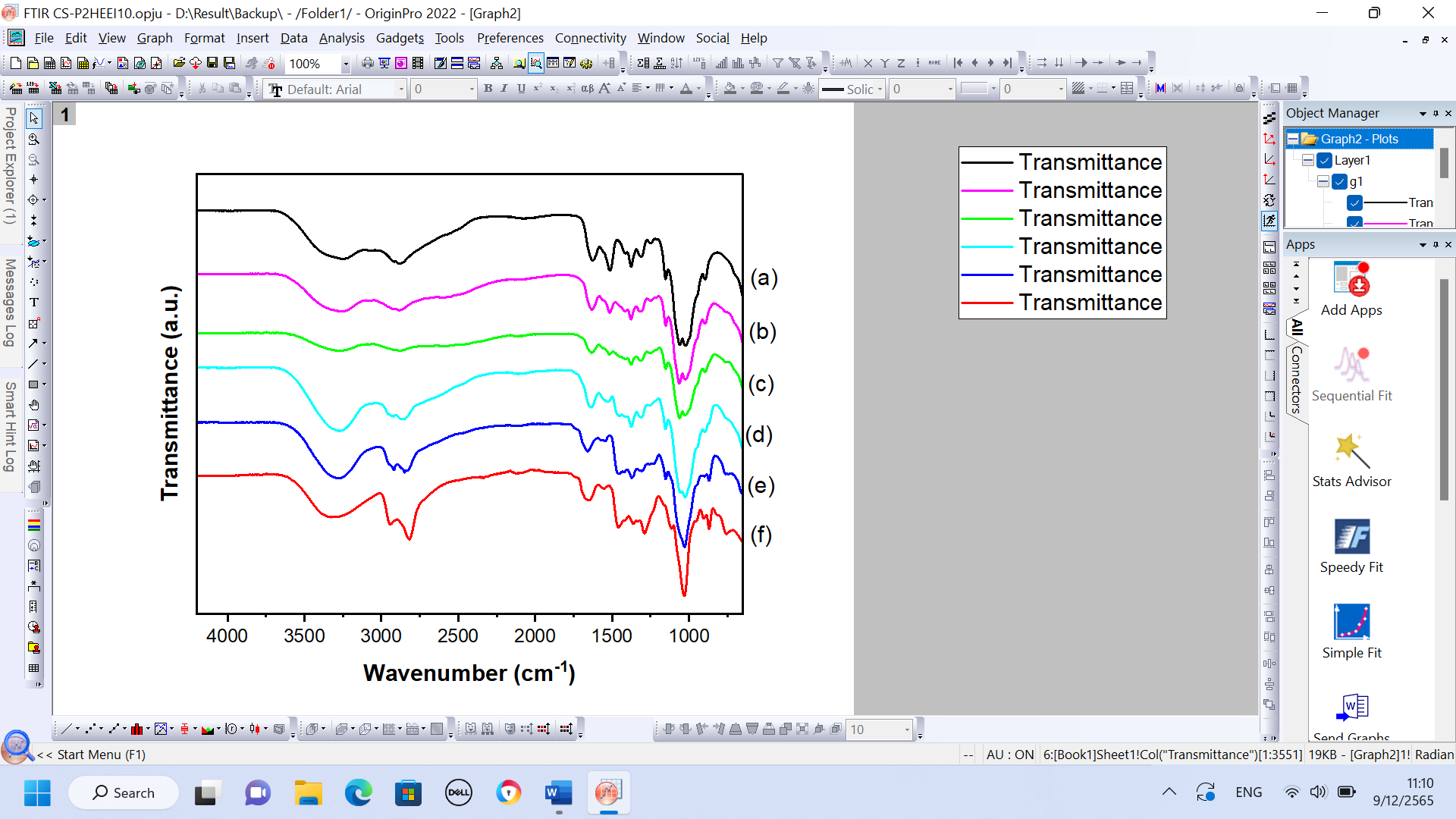


**Figure S5.** FTIR spectra of CHI (a), their blends (b, c, d and e) and P2HEEI (f). Content of P2HEEI in the blends: 20 (b), 40 (c), 60 (d) and 80 % (e).


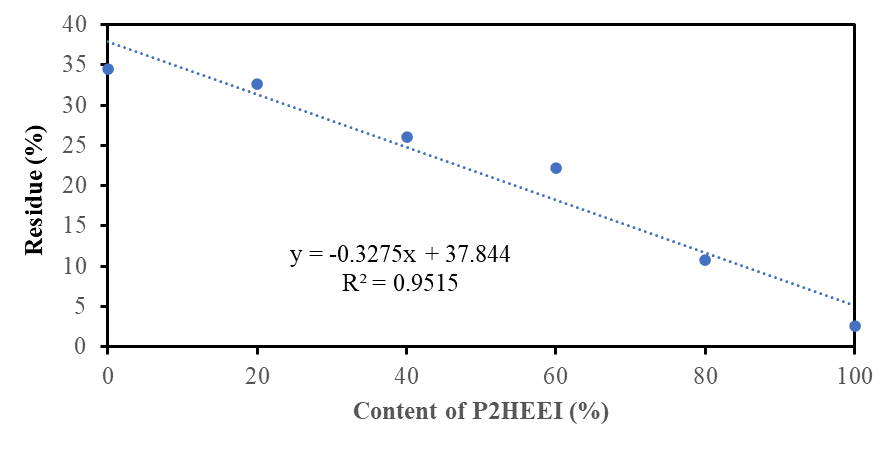


**Figure S6.** Correlation between residue and amount of P2HEEI in CHI/P2HEEI blends.

**Table S1.** thickness of films.

| Films | Thickness (mm) |
| --- | --- |
| CHI (100) | 0.06 ± 0.01 |
| CHI/P2HEEI (80:20) | 0.06 ± 0.01 |
| CHI/P2HEEI (60:40) | 0.07± 0.02 |
| CHI/P2HEEI (40:60) | 0.07 ± 0.01 |
| CHI/P2HEEI (20:80) | 0.07 ± 0.01 |


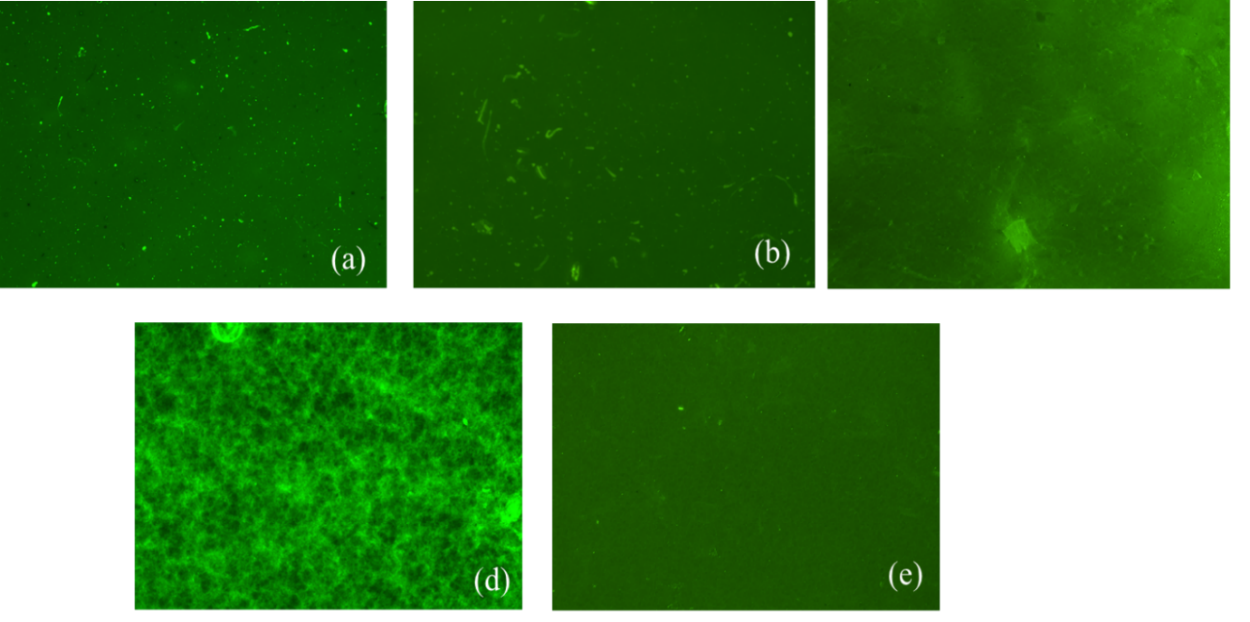


**Figure S7.** SEM images of film surfaces (A) and cross-section (B) of CHI (a) and their blends (b, c, d, and e). Content of P2HPEEI in the blends: 20 (b), 40 (c), 60 (d) and 80 % (e).

**
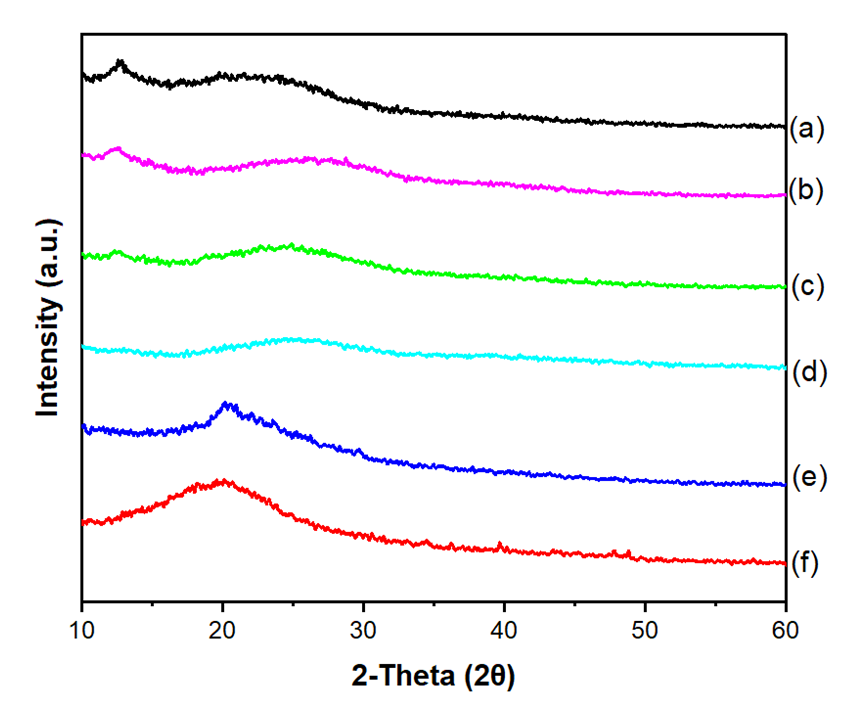
**

**Figure S8.** X-ray diffractograms of CHI (a), their blends (b, c, d and e), and P2HEEI (f). Content of P2HEEI in the blends: 20 (b), 40 (c), 60 (d) and 80 % (e).


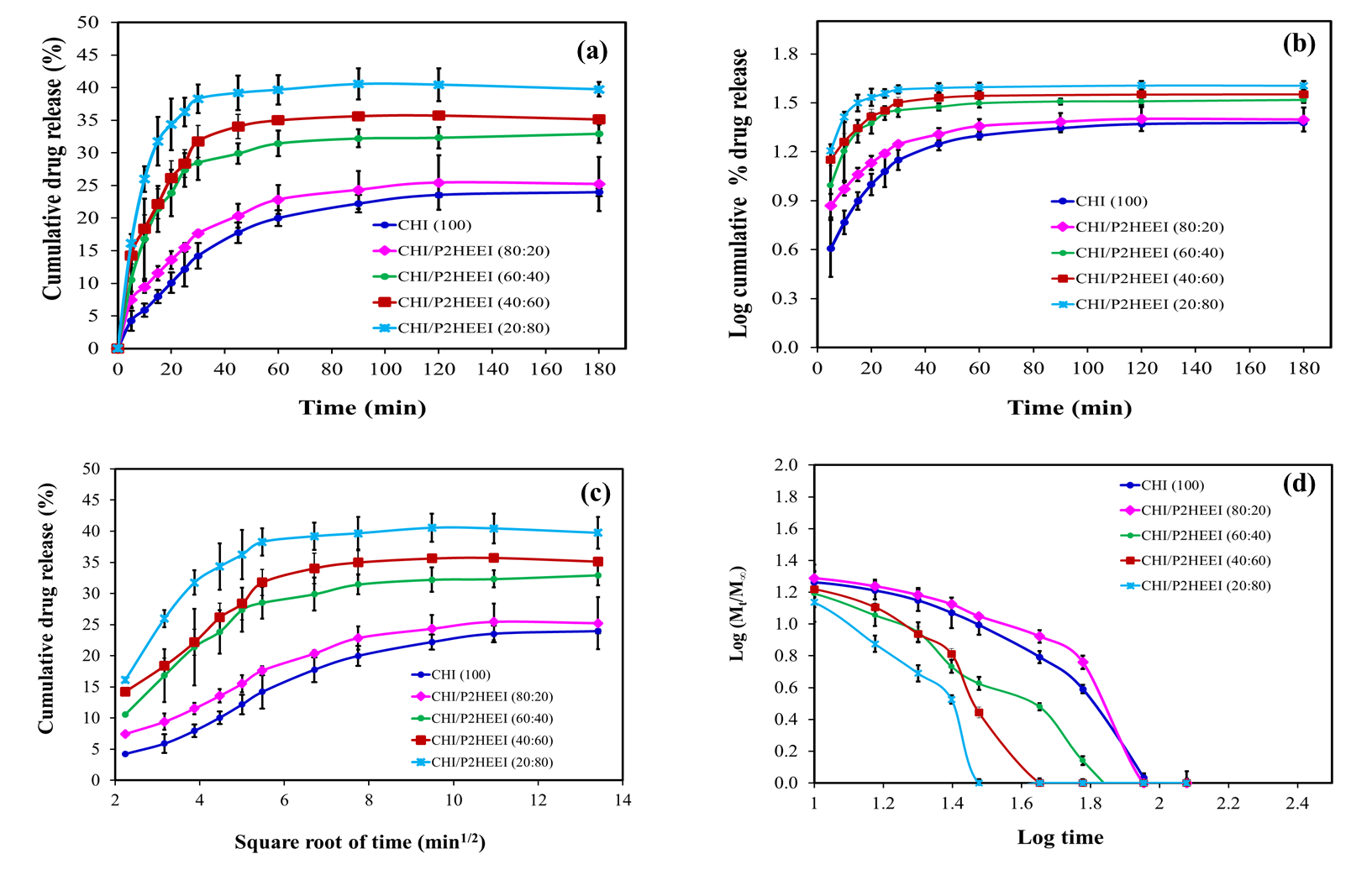


**Figure S9.** Drug release kinetic plots: Zero order (a), First order (b), Higuchi (c) and Korsmeyer–Peppas plot (d).

**Table S2.** Drug release kinetics (R^2^ value) for drug loaded films of CHI and blends with P2HEEI.

| CHI: P2HEEI blend films (%) | R^2^ value | | | | |
| --- | --- | --- | --- | --- | --- |
|  | **Zero order** | **First order** | **Higuchi** | **Korsmeyer–Peppas** | |
| 100:0 | 0.7382 | 0.6045 | 0.9016 | | 0.7749 |
| 80:20 | 0.6514 | 0.6003 | 0.8697 | | 0.7773 |
| 60:40 | 0.4512 | 0.3902 | 0.6891 | | 0.9500 |
| 40:60 | 0.4386 | 0.4259 | 0.6913 | | 0.8827 |
| 20:80 | 0.3246 | 0.2871 | 0.5408 | | 0.8576 |


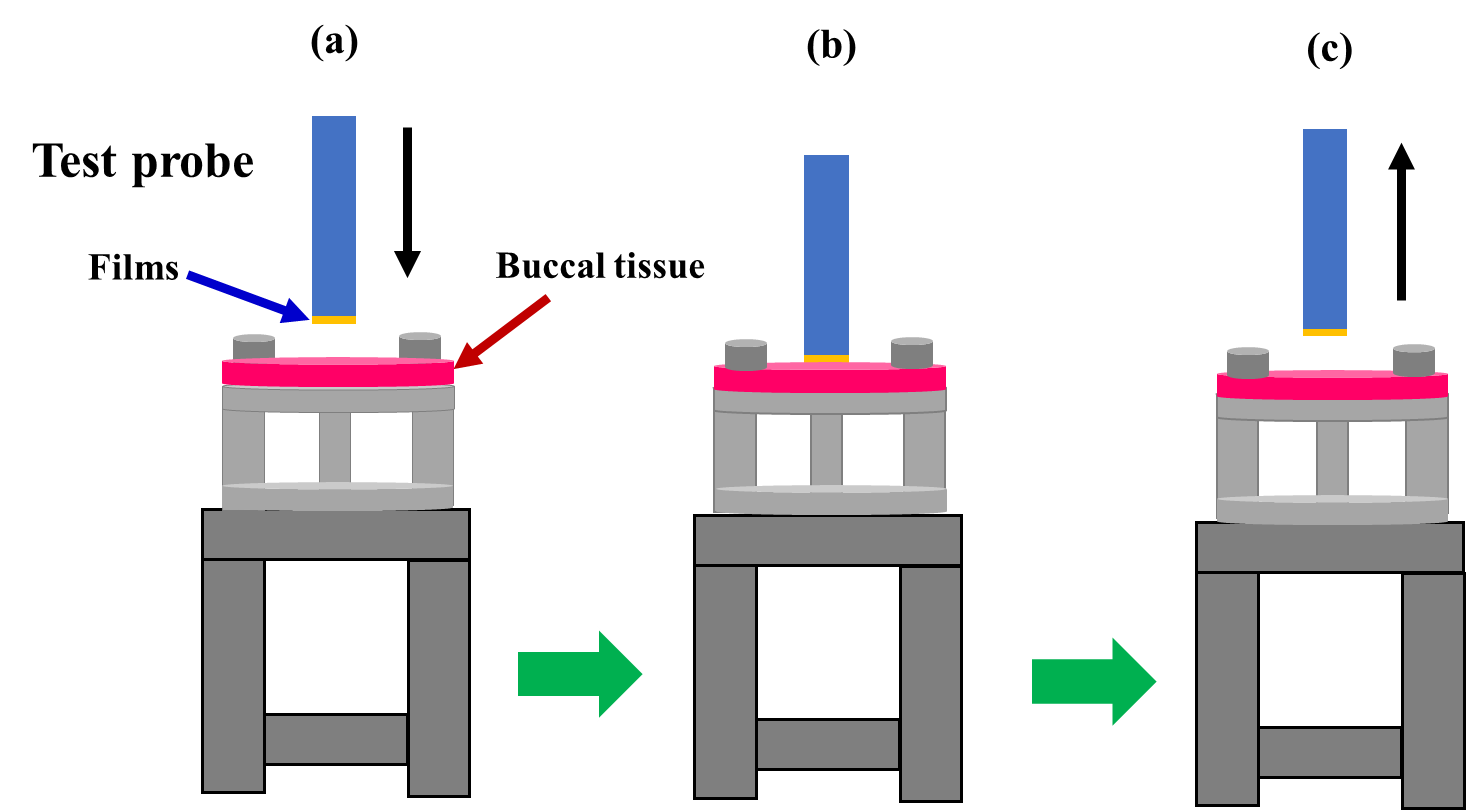


**Fig. S10.** Scheme process of *ex vivo* mucoadhesive test using texture analyzer with a mucoadhesive holder. (a) The probe with CHI and CHI/P2HEEI films was moved downward. (b) Film was attached to sheep buccal mucosa. (c) The probe is withdrawn at a specified rate.
